# In this issue

**Published:** 2023-02

**The utilization of artificial intelligence applications to improve breast cancer detection and prognosis**

Alsharif provides insight into the value of artificial intelligent (AI) tools combined with diverse imaging modalities in breast lesion detection and diagnosis. The shift from the conventional computer-aided detection (CAD) system to the advanced AI tools such as DL-CAD has the potential to reduce false-positive findings, increase diagnostic accuracy, improve radiologist performance, and assist with decision-making. Breast imaging faces challenges with the current increase in medical imaging requests and lesions that breast screening programs can miss. Solutions to improve these challenges are being sought with the recent advancement and adoption of artificial intelligent (AI)-based applications to enhance work ow efficiency as well as patient-healthcare outcomes. Using small datasets from the same source may raise concerns regarding the depth, quality, and representativeness of the images that are used to teach the AI-based applications and increase the possibility of bias and overfitting.

***see page 119***

