# Supplementary material for: In this issue
Source: Saudi Med J. 2023 Feb;44(2):117. (PMC9987697)
Supplement: Related texts

# Abstract: Original Articles

**Bilateral 4-quadrant laparoscopic-assisted transversus abdominis plane block reduces early postoperative pain after laparoscopic cholecystectomy.** ***A prospective, single-blind, randomized study***

Çevikkalp et al investigate 40 patients from Private Medicabil Hospital in Turkey the effcacy of bilateral 4-quadrant laparoscopic-assisted transversus abdominis plane (BLTAP) block in laparoscopic cholecystectomy (LC). The 1-hour visual analog scale (VAS) score at rest was lower in the BLTAP block group than in the standard analgesia (SA) and unilateral laparoscopic-assisted transversus abdominis plane (ULTAP) block groups. The change in VAS score was higher in the SA group than in the BLTAP block group. Bilateral 4-quadrant laparoscopic- assisted transversus abdominis plane block technique is more effective than standard analgesia, local anesthetic infiltration, and ULTAP block in preventing early postoperative pain after LC.

***see page 145***

**Figure:** Doyle’s bulge after the injection of local anesthetic.

**A prospective double-blinded randomized control trial comparing erector spinae plane block to thoracic epidural analgesia for postoperative pain in video-assisted thoracic surgery**

Hong et al compare 60 patients undergoing video-assisted thoracic surgery (VATS) received patient-controlled thoracic epidural analgesia (TEA) with a basal rate of 3 ml/hour (h), a bolus of 3 ml (Group E), or efficacies of erector spinae plane (ESP) block with programmed intermittent bolus infusions of 15 mL/3 h and a bolus of 5 ml (Group ES) for 2 postoperative days. Patients with continuous ESP block had a higher NRS score than those with TEA but no statistical difference at a specific time. Erector spinae plane block may be inferior to TEA for analgesia following VATS, but it could have tolerable analgesia and a better side effect profile than TEA. Therefore, it could be an alternative to TEA as a component of multimodal analgesia.

***see page 155***

# Abstract: Care Report

**Metastatic pancreatic cancer presenting with pulmonary arterial hypertension**

Alharbi et al report a 53-year-old male who was referred to the Pulmonary Medicine clinic for a dry cough for 6 weeks, associated with minimal sputum production which was yellowish in color without blood. Chronic cough is one of the most common symptoms encountered in respiratory clinics. It has a variety of differential diagnoses, and without a clear algorithmic approach, the final diagnosis can be overlooked. This case demonstrate the challenges of diagnosing pulmonary tumor thrombotic microangiopathy despite advancements in diagnostic imaging and the associated high fatality, despite advances in oncological treatments, mostly due to the aggressive behavior of the underlying malignancy and the relatively late diagnosis.

***see page 218***

**Figure:** Bilateral faint ground glass opacities with tree-in-bud appearance and mild bronchial wall thickening, more pronounced at the right lower lobe.
